# Supplementary material for: Holistic face recognition is an emergent phenomenon of spatial processing in face-selective regions
Source: Nat Commun. 2021 Aug 6;12:4745. doi: 10.1038/s41467-021-24806-1 (PMC8346587; doi:10.1038/s41467-021-24806-1)
Supplement: Supplementary file 1 — Supplementary Information [file 41467_2021_24806_MOESM1_ESM.pdf]

Holistic face recognition is an emergent phenomenon of spatial processing in face-selective regions

Supplementary Information

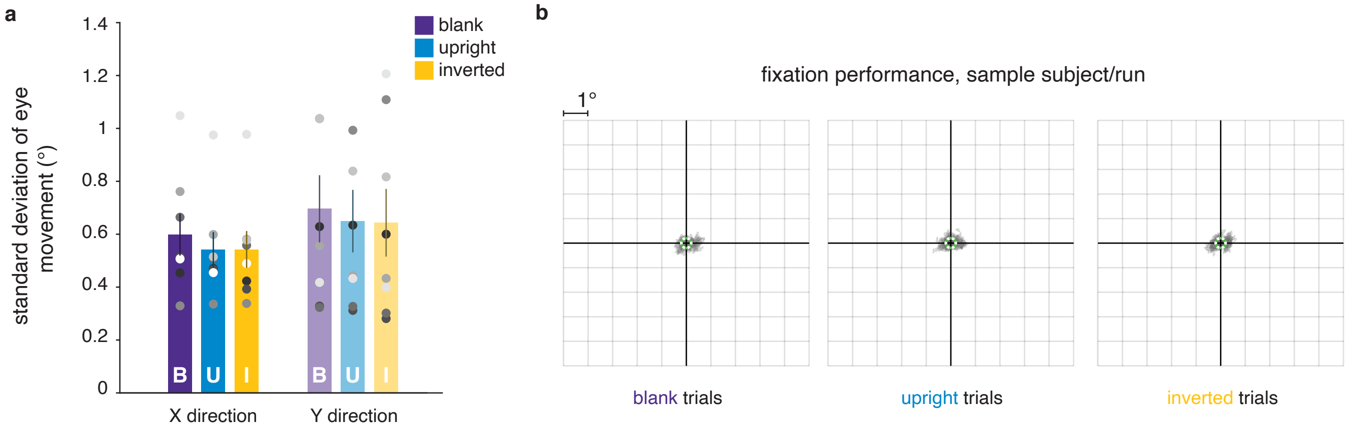

Supplementary Figure 1. Eye tracking data from pRF scan sessions. Participant eye position was monitored during fMRI scanning to ensure alertness and fixation performance. While interference from the scanner increased noise in the eye tracking measurements and not all participants were able to pass eye tracking calibration procedures, we recorded usable eye tracking data from 8 of 12 subjects, with an average of 77.5% (sd = 14.2%) of usable data across individual runs. Following standard preprocessing (blink and aberrant-spike removal, linear and quadratic trend removal), we compared the standard deviation of eye movements in the X and Y directions in the upright, inverted, and blank conditions. (**a**) Bars indicate the average (mean) standard deviations of eye movements around fixation for in the X and Y directions during blank (B), upright (U), and inverted (I) conditions. Each dot is data from a single participant. To evaluate whether the variability of eye movements varied with condition, we ran a two-way repeated measures ANOVA with factors of stimulus condition (upright, inverted, and blank) and direction of movement (horizontal X, vertical Y). There were no significant main effects of stimulus condition on the variability of eye movements ($F(1) = 0.20$, $p = 0.668$), nor an interaction with X/Y direction of eye movement ($F(2) = 0.76$, $p = 0.485$). There is a numerically larger variability in the Y direction compared to the X direction, perhaps attributable to blink traces, but this difference is not significant ($F(2) = 2.05$, $p = 0.166$). (**b**) Fixation performance in the three trial conditions for a sample subject and run, with the fixation aperture superimposed. The example illustrates that this participant's fixation was stable and did not vary substantially with condition.

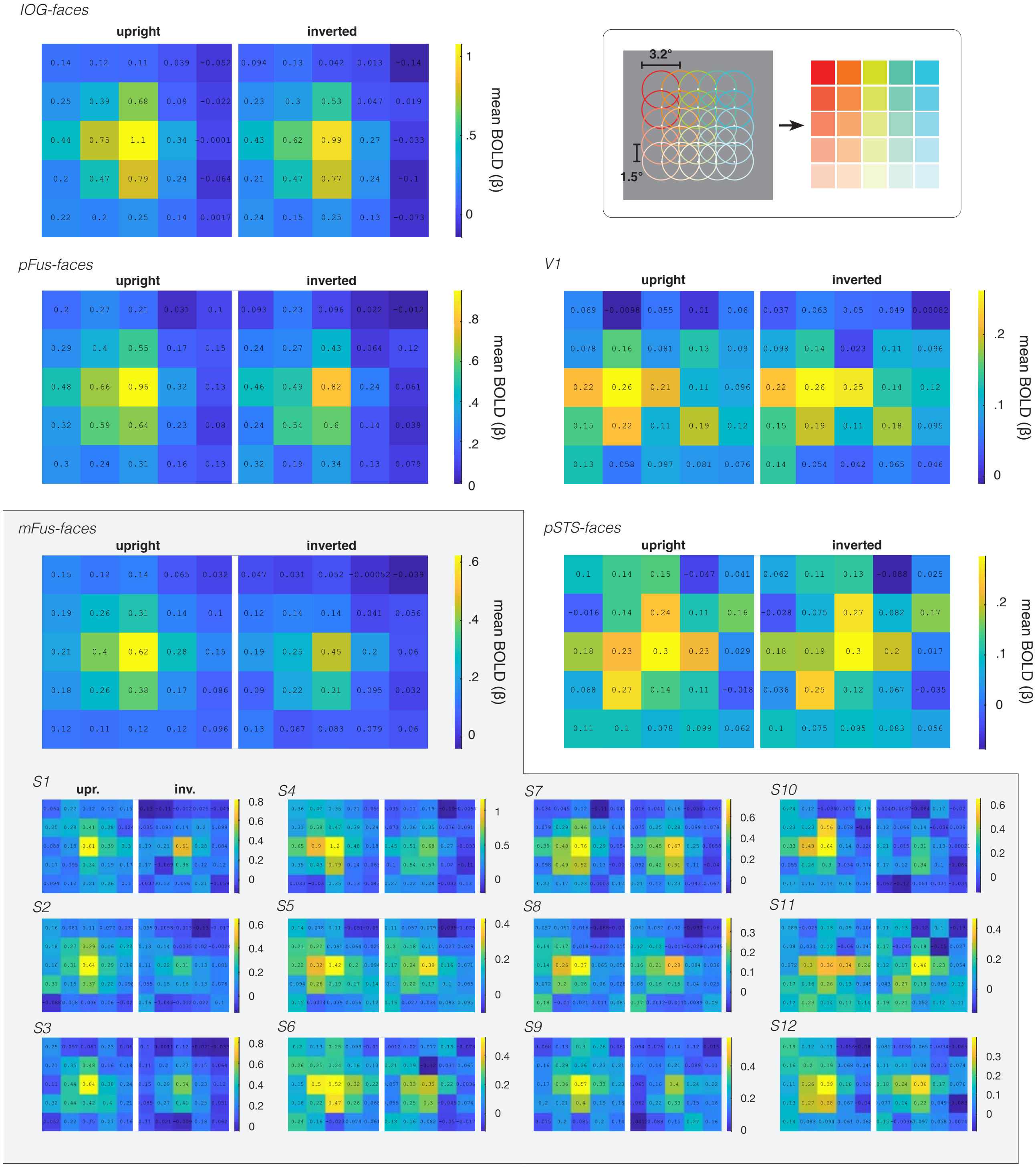

Supplementary Figure 2. Model-free mean fMRI responses in face-selective regions differ across space for upright and inverted faces. Mean response amplitudes (β estimates [% signal change]) for each ROI separately for upright (*left*) and inverted (*right*) conditions in each of the 25 locations in which faces were presented (see *top right panel*). Data are averaged across 12 participants. Individual subject (S1-S12) mean BOLD responses for mFus-faces are shown in the grey inset. These data provide evidence that differences in visual field coverage estimated from pRF modeling are not an outcome of modeling choices, but rather reflect the different measured responses across upright and inverted faces.

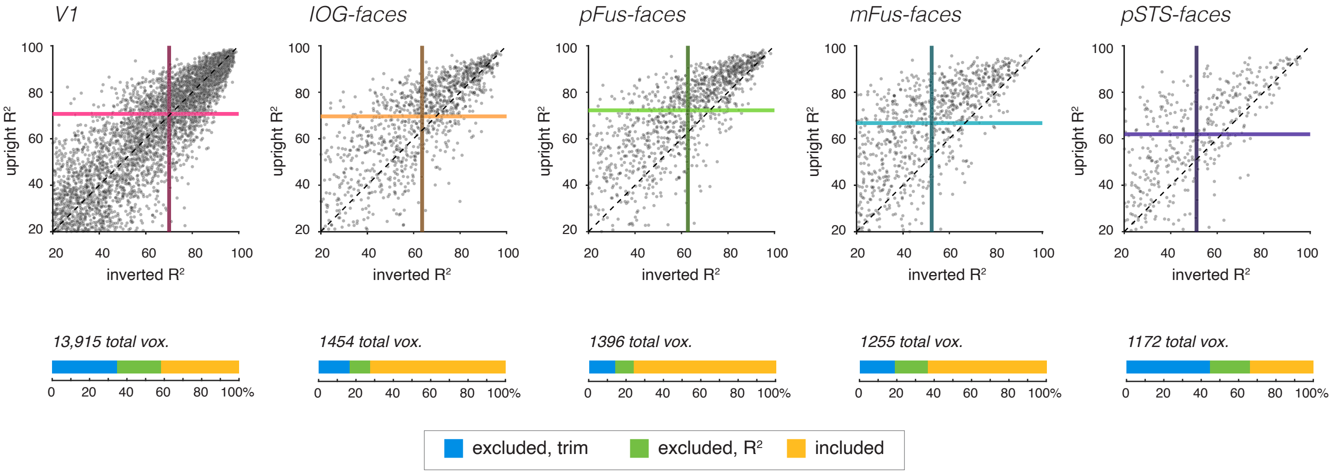

Supplementary Figure 3. Voxelwise comparison of model goodness-of-fit across upright and inverted faces. Scatterplots show the model goodness-of-fit (R^2) for each voxel in the data set for the upright vs. inverted face mapping conditions. Voxels are pooled across participants. Colored lines indicate the mean R^2 across voxels in the upright (horizontal, *light colors*) and inverted (vertical, *dark colors*) mapping conditions. The bottom panel illustrates the proportion of voxels excluded via our standard trimming procedure (see Methods: pRF model fitting), and subsequently by R^2 threshold. Model goodness-of-fit was equivalent across mapping conditions in V1, but was lower in the inverted face condition in all face-selective areas (full statistics in **Table S1**).

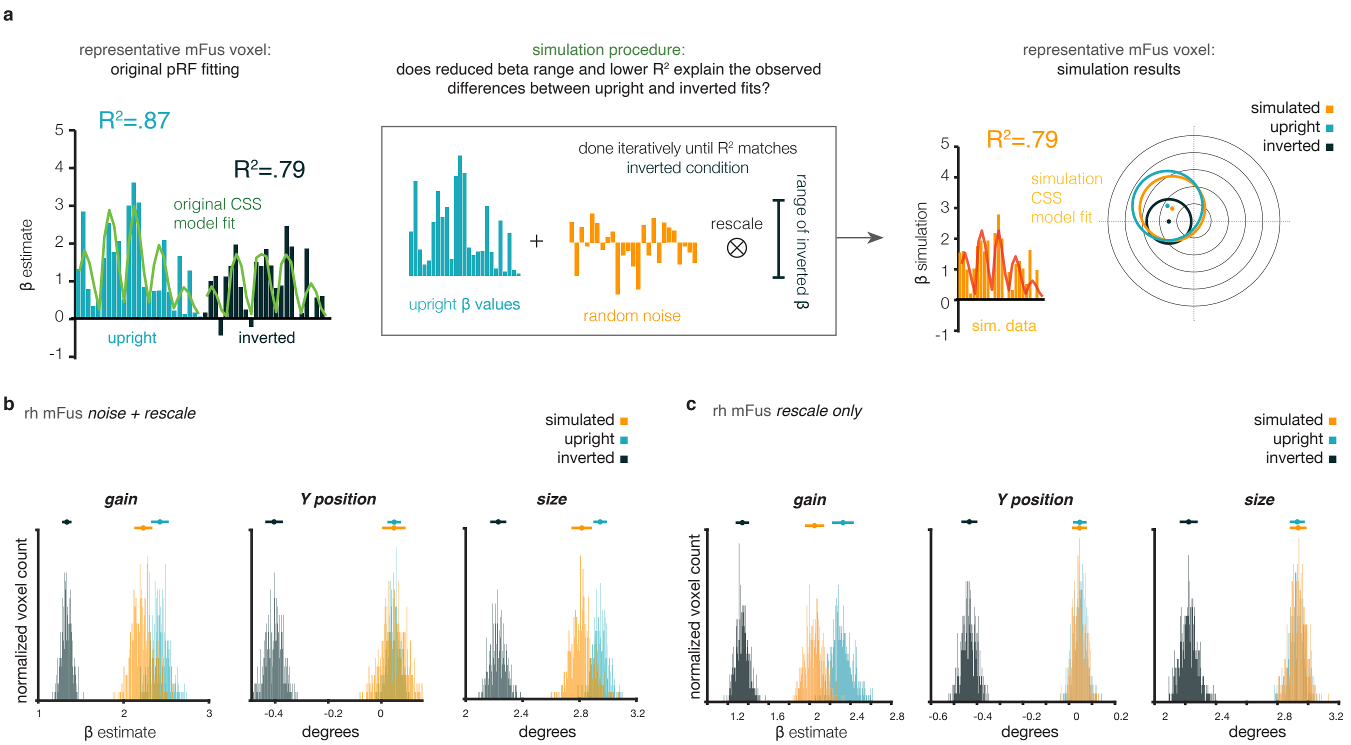

Supplementary Figure 4. Iterative noise simulation procedure and results. (a) Simulation procedure. To what degree do the observed differences in signal strength and R^2 between inverted and upright faces contribute to differences in pRF position and size estimates? To determine this, we ran a simulation over mFus-faces voxels: for each voxel, we took the 25 response amplitudes βs from the upright mapping condition (*light blue*), and then iteratively added random noise (noise step) until the simulated model fit R^2 was equal to the model R^2 in the inverted condition. Following this, we rescaled these noisy responses to match the range of the 25 Betas from the inverted mapping condition (*dark blue*; rescale step). In this example voxel, the simulated pRF fit (*orange*) much more closely resembles the upright than the inverted condition fits. **(b)** Bootstrapped parameter estimates for the rescaling + noise procedure described in **(a)**. While the simulated fits do show some differences in size and gain compared to upright pRFs, the properties of the simulated pRF estimates much more closely resembled the upright than inverted pRFs. Differences between upright and simulated pRFs are substantially smaller in magnitude than what we observe for inverted faces, despite matching R^2 and response levels (βs). Horizontal line markers above distributions indicate median and 68% confidence intervals of the parameter estimates. **(c)** Results of an additional simulation which was the same as (b) except that we omitted the noise step. Changing the response amplitude has minimal effect on the size or position of pRFs. Together, these simulations allow us to conclude that differences in model goodness-of-fit and weaker signal in response to inverted faces cannot fully account for the observed differential pRF estimates for upright and inverted faces in the main experiment.

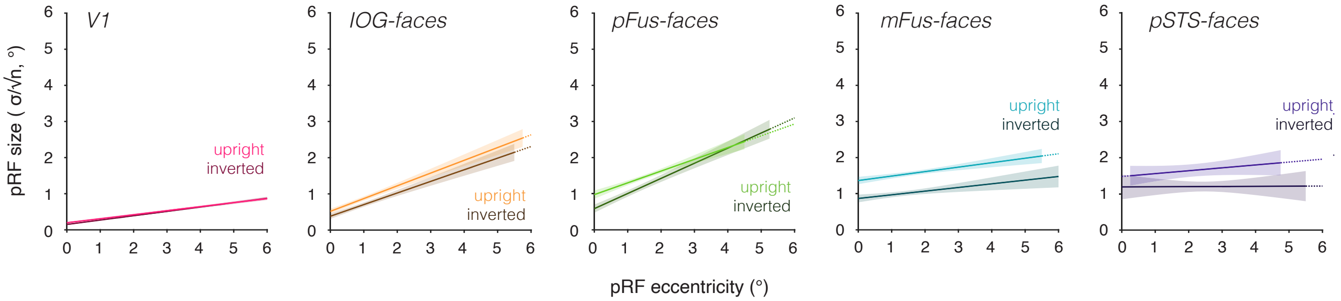

Supplementary Figure 5. Inverted-mapped pRFs are smaller in face-selective regions across eccentricity. Size by eccentricity relationship in each ROI is estimated for each individual participant, then averaged across participants (N=12) to provide an estimate of between-participant variability. Lines are generated by fitting least-squares lines to individual participant data and then averaging these fits. The line is subsampled at 0.25°, and shaded regions indicate ± SEM of the estimated model across participants, at eccentricities containing > 0 voxels in each individual participant. Decreased size for inverted-mapped pRFs is most apparent in mFus-faces, but is also evident in pFus- and pSTS-faces. We observed no reliable size differences across participants in V1 or IOG-faces. Full statistics in **Table S2**.

Supplementary Figure 6. Size changes across upright and inverted faces are driven primarily by differences in the estimated pRF sigma (σ) parameter. The compressive spatial summation (CSS) model quantifies pRF size as $\sigma/\sqrt{n}$, where $0 < n < 1$ is the estimated compressive exponent parameter. Here, we plot the exponent **(a)** and sigma **(b)** parameters separately in order to gain insight into the nature of pRF changes across upright and inverted conditions. *Markers:* mean across participants (N=12) for upright faces (*light gray*) and inverted faces (*dark gray*). *Error bars*: ± SEM across participants. Results show that across participants changes in pRF size with face inversion are driven by reduced σ estimates, not compression n. Significance is demonstrated via 2-way repeated-measures ANOVA across bilateral face-selective areas (right tables). While the exponent estimate n trends lower in some face-selective regions in response to inversion, smaller exponents yield larger pRF size ($\sigma/\sqrt{n}$) estimates. Thus, the reduction in σ estimates outweighs the reduction in exponent estimates, leading to overall smaller pRF sizes in the inverted condition (**Figure 2**).

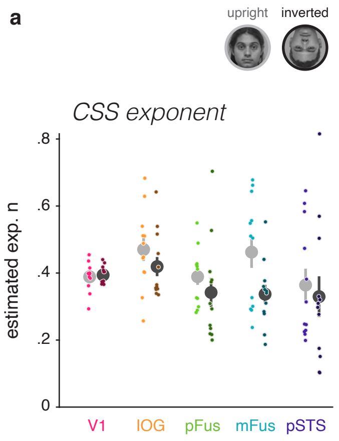

CSS exponent		
significant main effects:		
none		
significant interactions:		
none		
not significant:		
ROI	F(3) = 1.85	p = 0.158
condition	F(1) = 2.69	p = 0.129
ROI x condition	F(3) = 1.36	p = 0.271

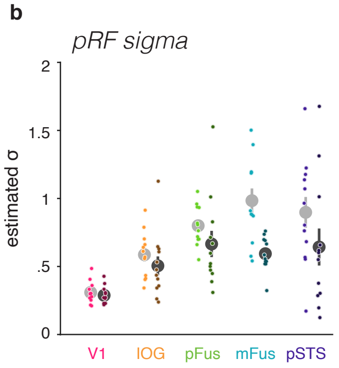

estimated σ		
significant main effects:		
condition	F(1) = 7.59	p = 0.019
significant interactions:		
ROI x condition	F(3) = 3.32	p = 0.032
not significant:		
ROI	F(3) = 2.85	p = 0.052

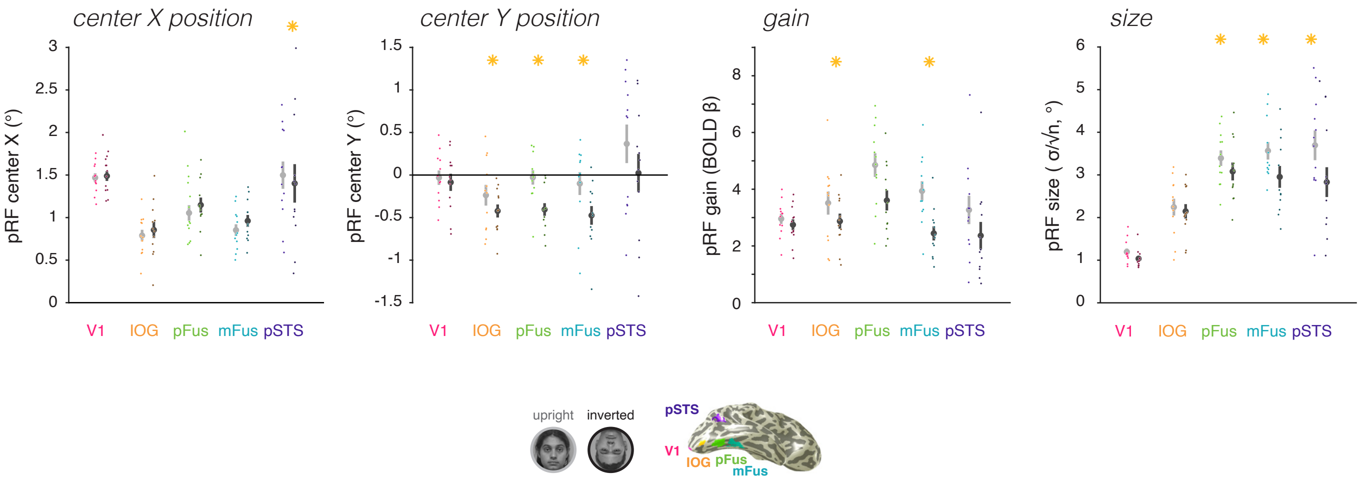

Supplementary Figure 7. pRF parameters for upright and inverted faces for a linear pRF model. Estimated parameters using a linear pRF model with 4 parameters: **(a)** center X, **(b)** center Y, **(c)** gain, and **(d)** size σ. Plotting follows **Figure 2**. While the inclusion of a compressive exponent n in the pRF model improves model accuracy in higher-level visual areas including the face-selective regions, the use of this particular model is not absolutely necessary for the current study's goal of estimating pRF parameters of interest (e.g., position, size). To demonstrate this point, here we show results obtained using a linear pRF model (exponent n =1), closely follow the pattern of results reported from the CSS model. In particular, a 2-way repeated-measures ANOVA across bilateral face-selective areas reveals a significant main effect of mapping condition (upright/inverted faces) on the pRF Y position ($F(1,10) = 13.43$, $p = 0.004$), σ estimate of pRF size ($F(1,10) = 45.3$, $p = 5.1\times10^{-6}$), and gain $F(1,10) = 67.12$, $p = 9.6\times10^{-6}$). Full statistical results in **Table S3**.

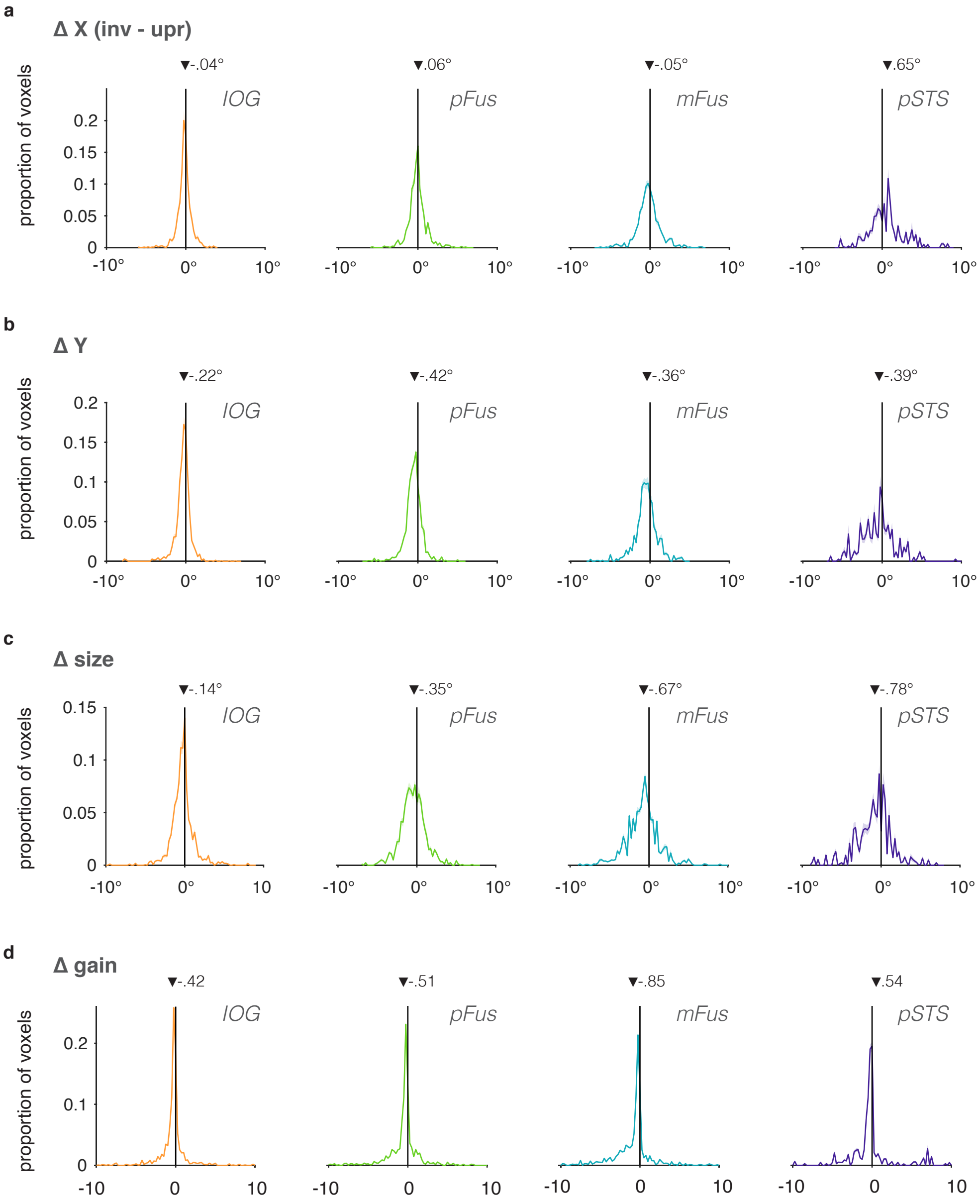

Supplementary Figure 8. Distributions of voxelwise differences in pRF parameters across mapping with inverted and upright faces (inverted minus upright). Bins are 0.25° wide, and shaded error marks ± SEM across participants (N = 12). *Triangle markers* indicate mean value across subjects.

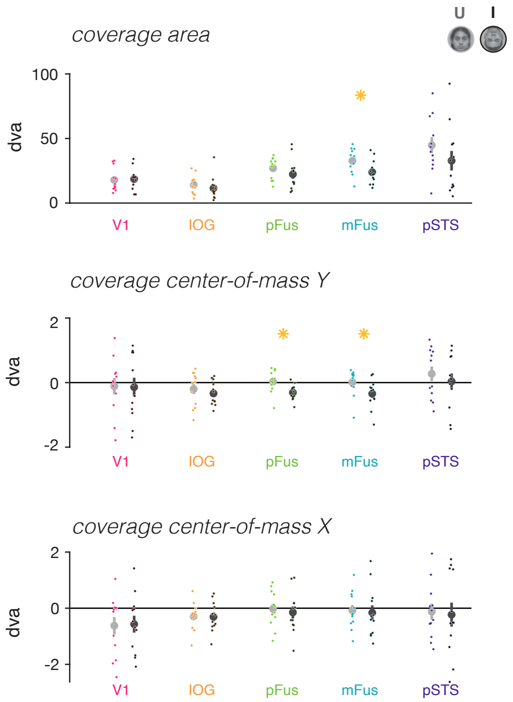

Supplementary Figure 9. Coverage metrics across participants. We estimated for each ROI and participant the area of visual field coverage spanned by pRFs of each ROI and the center-of-mass of this coverage. Area (*top*) was computed for the region at full-width-half-maximum of the coverage plots shown in **Figure 5**. The center of mass is taken over the same region, and has two parameters: a vertical Y coordinate (*middle*) and horizontal X coordinate (*bottom*). *Markers:* mean across participants (N=12) for upright faces (light gray) and inverted faces (dark gray). *Error bars:* ± SEM across participants (N = 12). *Dots:* individual participant metrics. *Asterisks:* significant differences between upright and inverted face conditions at $p < .05$, post-hoc, paired two-sided t-test.

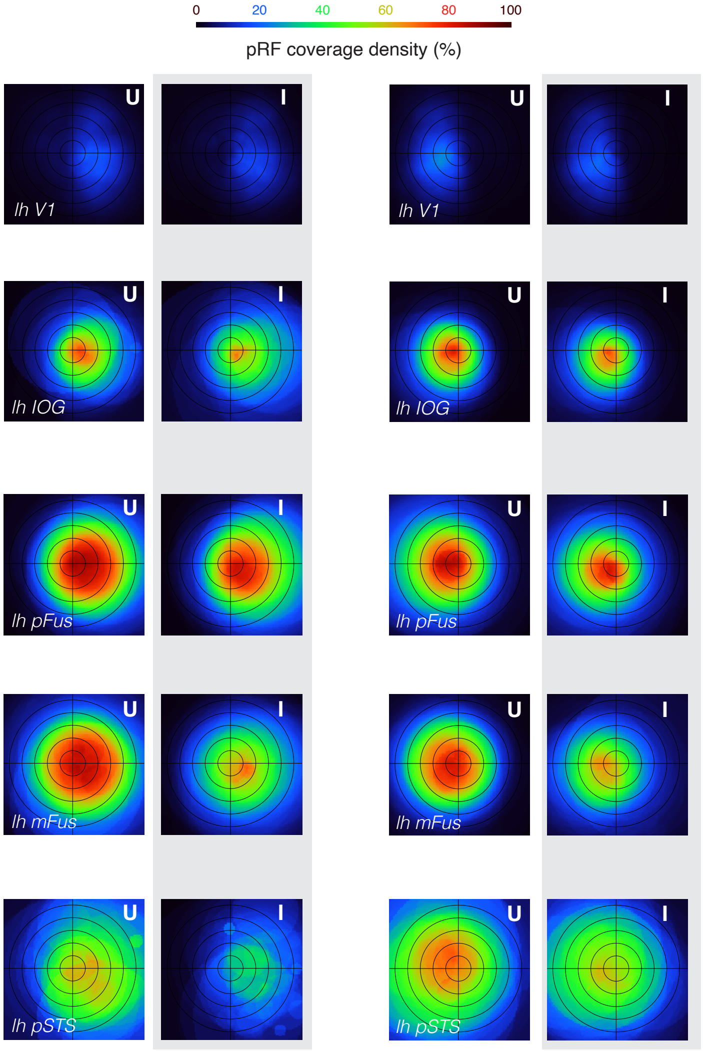

Supplementary Figure 10. Average pRF visual field coverage density across participants by ROI and hemisphere. Plotting conventions follow **Figure 5**. Visual field coverage density approximates the proportion of pRFs that overlap a given region in space. Mapping with upright faces (U) replicates previously reported properties of coverage in face-selective regions, including a strong contralateral representation of the fovea in pFus- and mFus-faces[1,2], and less foveal bias in pSTS-faces than in the ventral face-selective regions[3]. Mapping with inverted faces (I) yields significantly smaller full-width-half-max coverage area in lh-mFus, rh-mFus, lh-pSTS (posthoc two-sided t's(11) > 2.30, p's < 0.044) and a significant downward shift of the center-of-mass of the coverage in lh-pFus, rh-pFus, rh-IOG (posthoc two-sided ts(11) > 2.33, ps < 0.041). There were no significant differences in the horizontal location of the center-of-mass in any region and no significant changes to any of these parameters in either left hemisphere (lh) or right hemisphere (rh) V1.

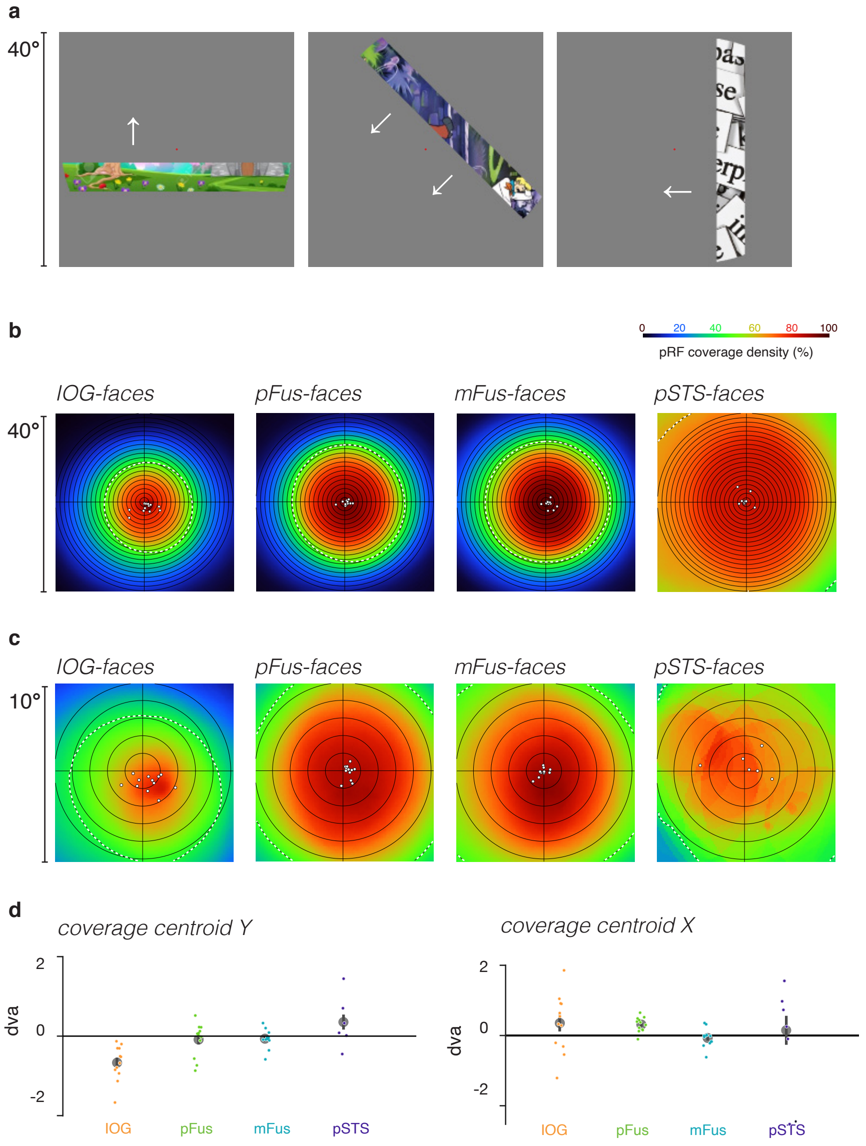

Supplementary Figure 11. pRF mapping with cartoon stimuli yields pRF coverage consistent with upright-face mapping. All 12 participants of the current study also took part in a separate pRF mapping experiment that used different mapping stimuli, mapping timing, and fitting procedures[3], allowing us to evaluate whether any non-optimal mapping stimulus would yield a similar reported pattern of results as inverted faces, or whether changes in pRF properties and coverage in face-selective regions are specific to face inversion. For brevity, here we refer to 'Toonotopy data' as the subset of widefield data from Finzi et al.[3] from the participants of the current study, analyzed using the same participant-wise ROIs as in the current study. Unlike the current study, for the Toonotopy data we fit the CSS pRF model for the mean timecourse of each voxel (procedure: http://cvnlab.net/analyzePRF) rather than to estimated βs for discrete mapping stimuli. The Toonotopy widefield mapping stimulus extended to 20° eccentricity; to better compare the Toonotopy data to the current study, we analyzed both the full widefield Toonotopy datasets of the 12 subjects, and also a subset of the data matched to the spatial extent of the mapping stimuli in the current study (eccentricity < 5°, size < 10°). **(a)** Widefield Toonotopy mapping stimuli were comprised of rapidly-presented cartoon frames (8Hz presentation) within a bar aperture that swept the visual field in 8 directions, and covered the central 40° of the visual field over the course of an fMRI run. **(b)** For the 12 participants in the current study, the average pRF

coverage in face-selective ROIs from the full widefield Toonotopy data. As in Figure 5, *white contour:* the average FWHM of coverage density. *White dots:* individual participants' FWHM center-of-mass position. As expected, the widefield nature of the Toonotopy stimuli of Finzi and colleagues[3] allow us to measure pRFs beyond the ~10° extent of the mapping stimuli used in our main experiment. To draw better comparison to the current study, **(c)** presents equivalent coverage plotting of the subset of subjects' pRFs in the widefield Toonotopy for the data matched to the ~10° extent of the mapping stimuli of the current study. Cross-participant metrics of this coverage in the face-selective regions are shown in **(d)**. While we cannot quantitatively compare visual field coverage area between the two studies due to the differing spatial extent of the mapping stimuli, we see qualitatively similar coverage in response to the Toonotopy mapping stimuli and the upright face mapping in the current study. That is, the visual field coverage was foveally biased in ventral face-selective regions and progressively increased from IOG- to pFus- to mFus-faces. Critically, we did not find evidence of a downward shift in the center-of-mass of visual field coverage in pFus- and mFus-faces for the Toonotopy data as we observed with face inversion. *Error bars*: ± SEM across 12 participants.

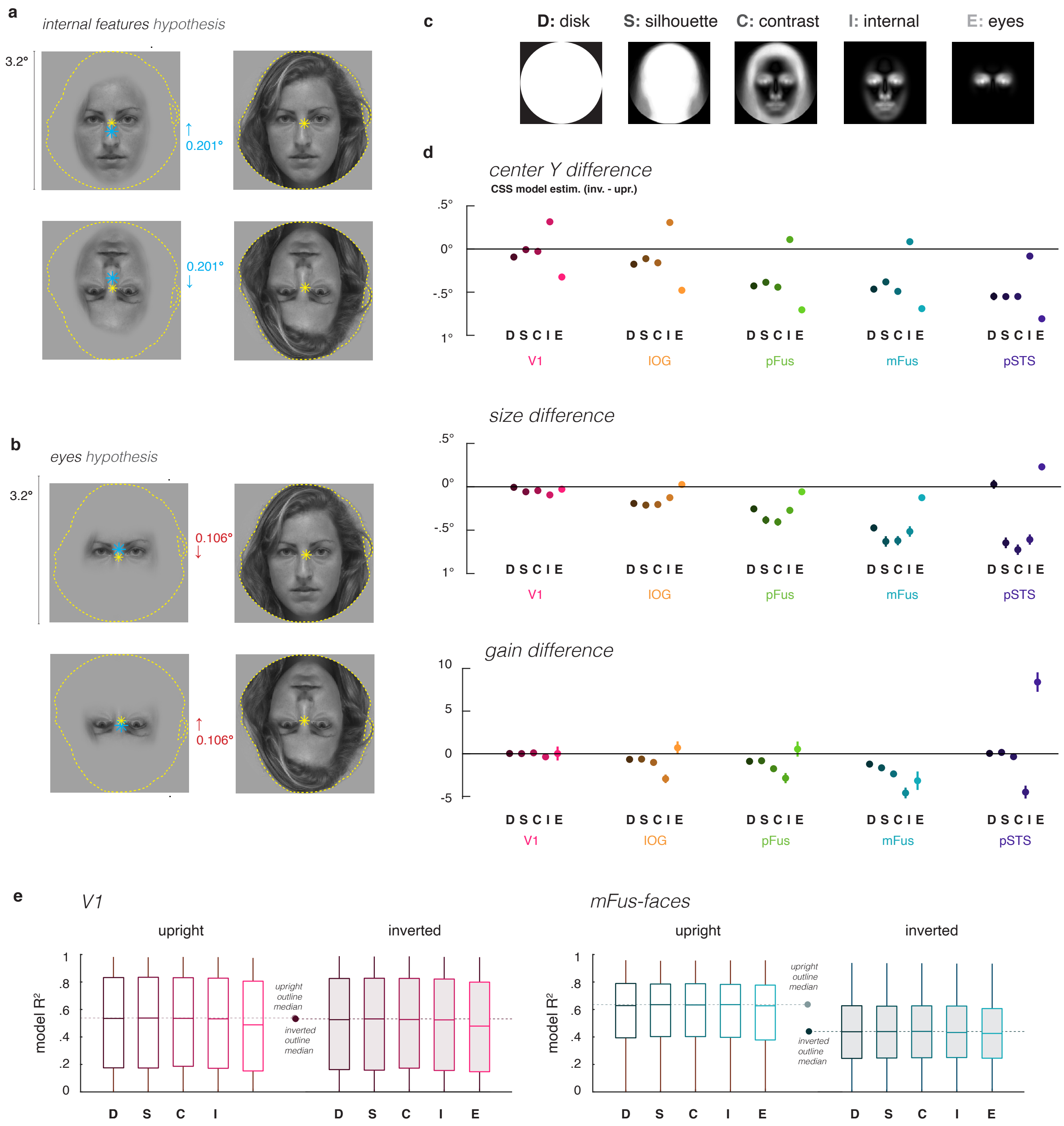

Supplementary Figure 12. Comparing results across pRF models with different codings of the stimulus to determine the relationship between internal feature location, stimulus coding, and pRF estimates. In a schematic, we present two hypotheses that examine how the location of internal features **(a)** or eyes alone **(b)** may contribute to the positional shifts in pRFs due to face inversion. In the pRF model, each individual face location is coded as a binary silhouette of the full-face image that participants saw, here shown via *yellow dashed line*. Because pRF model fitting relies on computing the dot product of a hypothesized Gaussian pRF and this binary image, we can infer that the center-of-mass of the binary silhouette is an important factor in determining the estimated pRF position. The weighted centroid of the binary silhouette for the sample image is marked by *yellow asterisks* in all panels. To account for the observed shift in Y position in response to face inversion, we evaluated the **(a)** I*nternal features hypothesis:* the position of the

internal face features, rather than the full face image, drives spatial responses in face-selective regions. This can be approximated by calculating the weighted centroid of the internal features of this face for the upright and inverted conditions (left panel, *blue asterisks*). Correspondingly, if the internal features rather than the entire face determine the location of the pRF in both the upright and inverted mapping conditions (blue), our implemented pRF model (yellow) will yield position estimates ~0.2° above the location of the internal features of the face in the upright condition, and ~0.2° below in the inverted condition. The overall consequence of these offsets would be a ~0.4° lower pRF estimate in the inverted condition than in the upright condition, which is similar to the magnitude and directionality of effects we observed in pFus- and mFus-faces. **(b)** *Eyes hypothesis:* the position of the eyes alone, rather than the full face image, drives spatial responses in face-selective regions. This can be approximated by calculating the weighted centroid of the eyes of this face for the upright and inverted conditions (right panel, *blue asterisks*). Correspondingly, if the location of the eyes within the mapping stimulus drives spatial responses in a voxel, we would expect to see (i) smaller difference between the upright- and inverted-mapped pRF estimates, as the centroid of the eye region of our face images (blue asterisk) is typically well-aligned with the centroid of the binary silhouette (yellow), and (ii) inverted-mapped pRFs that are shifted upward ~0.2° relative to the upright-mapped condition. This is a poorer match to the effects of face inversion observed experimentally; neither manipulation predicts the observed differences in pRF gain or size. **(c)** These hypotheses are explicitly tested by re-coding the location of experimental stimuli in four new ways: as a [D] circular disk aperture, [C] contrast image corresponding to the absolute contrast of the mapping faces, [I] internal features of the contrast images, and [E] the eyes of the contrast images. The silhouette outline binary mask used in the main analyses is labeled as [S] (**Figure 1b**). In all cases, the pRF model was fit using the same methods as previously described across data from all 12 participants in V1 and face-selective IOG-, mFus-, and pFus-faces. We expect that more complex feature sensitivity to the eyes or other internal features to be more prominent in the face-selective regions than in early visual cortex. In (c), we show bootstrap estimates of the difference in pRF parameters between upright and inverted mapping for these 5 stimulus codings. To compare across models, bootstrapping is done on voxels in face-selective ROIs with R^2 in both conditions > 0.5. As predicted, we see a strong relationship between the estimated pRF center Y and the center-of-mass of the coding stimuli. While the internal features (I) coding appears to account for a large part of the Y-shift in mFus- and pFus-faces, it also introduces a shift of equivalent magnitude in IOG-faces and V1. Critically, we see in the raw data (**Supplementary Figure 2**) that responses in these regions are largely equivalent for upright and inverted faces, making these apparent differences in pRF parameters inconsistent with the measured data. Moreover, we note that the use of internal features [I] coding still does not account for the differences in pRF size and gain observed across upright and inverted faces. **(d)** Model goodness-of-fit (R^2) for V1 and mFus-faces for each of the new stimulus coding implementations. Here, voxel R^2 is not thresholded. We see no evidence that the internal-features model explains more variance than the implemented coding. Thus, from the perspective of model goodness-of-fit comparisons, there is no reason to favor this stimulus coding over others. For comparison, *dotted lines* mark the median R^2 of the main manuscript model fitting using coding [S]. Boxplots depict the median (50th), 25th, and 75th percentiles; whiskers extend to the minimum and maximum data points. All individuals depicted provided informed consent for the publication of their likeness.

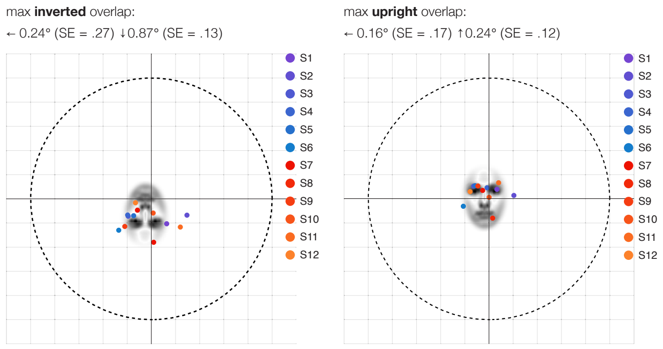

Supplementary Figure 13. Positions in the visual field yielding maximal pRF overlap with internal features of upright and inverted faces at center for each of our participants*.* As described in Figure 6, we sought to determine the position in space at which the internal features of a 3.2° upright face (*right panel*) maximally overlapped with upright-mapped pRFs, and the position in space at which the internal features of an inverted face (*left panel*) maximally overlapped with inverted-mapped pRFs. The bootstrapping simulation used to determine the positions at which faces were shown in the behavioral experiment was based on pooled preliminary data from the first 6 participants in the experiment (S1-6, *cool colors*). We repeated the simulation procedure on an individual-participant basis (1000 draws per participant, 80% of voxels sampled with replacement), including the participants whose pRF data was collected after the initial simulation (S7-12, *warm colors*). *Dots:* bootstrapped estimates for individual participants. *Face images* in each panel are shown at the mean maximal overlap position in each condition. Overall, while some inter-participant variability is present, the mean positions determined across all participants align well with the position used in the behavioral experiment, such that maximal overlap for inverted faces occurs in the lower left visual field, while maximal overlap for upright faces occurs is more central and trends slightly to the upper left.

Supplementary Table 1. Results of three-way repeated measures analyses of variance (ANOVA) with factors of hemisphere (right/left), region of interest (IOG-/pFus-/mFus-/pSTS-faces), and condition (upright/inverted) for each pRF mean parameter estimate across participants. One participant had insufficient lh-pSTS data (0 voxels above threshold) and was excluded from this analysis, leaving N = 11. Related to **Figure 2.**

center position Y estimate		
significant main effects:		
condition	$F(1,10) = 8.82$	$p = 0.014$
significant interactions:		
none		
not significant:		
hem	$F(1,10) = 0.10$	$p = 0.76$
ROI	$F(3,30) = 0.10$	$p = 0.68$
hem x ROI	$F(3,30) = 0.55$	$p = 0.65$
hem x condition	$F(1,10) = 2.61$	$p = 0.14$
ROI x condition	$F(3,30) = 0.83$	$p = 0.49$
hem x ROI x condition	$F(3,30) = 0.67$	$p = 0.58$

center position X estimate		
significant main effects:		
hem	$F(1,10) = 375.60$	$p < 1.0 \times 10^{-8}$
significant interactions:		
hem x ROI	$F(3,30) = 4.49$	$p = 0.010$
not significant:		
ROI	$F(3,30) = 2.15$	$p = 0.11$
condition	$F(1,10) = 0.31$	$p = 0.59$
hem x condition	$F(1,10) = 1.16$	$p = 0.31$
ROI x condition	$F(3,30) = 0.39$	$p = 0.76$
hem x ROI x condition	$F(3,30) = 0.056$	$p = 0.98$

size estimate ($\sigma / \sqrt{n}$)		
significant main effects:		
ROI	$F(3,30) = 7.13$	$p = 9.4 \times 10^{-4}$
condition	$F(1,10) = 56.07$	$p = 2.1 \times 10^{-5}$
significant interactions:		
hem x ROI	$F(3,30) = 3.36$	$p = 0.032$
ROI x condition	$F(3,30) = 7.53$	$p = 6.7 \times 10^{-4}$
not significant:		
hem	$F(1,10) = 0.01$	$p = 0.92$
hem x condition	$F(1,10) = 1.18$	$p = 0.30$
hem x ROI x condition	$F(3,30) = 2.64$	$p = 0.068$

gain estimate		
significant main effects:		
none		
significant interactions:		
ROI x condition	$F(3,30) = 4.66$	$p = 0.0086$
not significant:		
hem	$F(1,10) = 2.77$	$p = 0.13$
ROI	$F(3,30) = 0.61$	$p = 0.62$
condition	$F(1,10) = 2.07,$	$p = 0.18$
hem x ROI	$F(3,30) = 0.75$	$p = 0.53$
hem x condition	$F(1,10) = 0.052$	$p = 0.82$
hem x ROI x condition	$F(3,30) = 0.36$	$p = 0.78$

model R^2		
significant main effects:		
ROI	$F(3,30) = 17.59$	$p = 8.9x10^{-7}$
condition	$F(1,10) = 80.36$	$p = 4.3x10^{-6}$
significant interactions:		
ROI x condition	$F(3,30) = 7.47$	$p = 7.1x10^{-4}$
not significant:		
hem	$F(1,10) = 0.19$	$p = 0.67$
hem x ROI	$F(3,30) = 1.13$	$p = 0.35$
hem x condition	$F(1,10) = 1.28$	$p = 0.28$
hem x ROI x condition	$F(3,30) = 0.23$	$p = 0.87$

Supplementary Table 2. Results of two-way repeated measures ANOVA with factors of ROI (IOG-/pFus-/mFus-/pSTS-faces) and condition (upright/inverted) on the relationship of size and eccentricity in bilateral face-selective areas (related to **Supplementary Figure 3**) across participants (N=12).

fit line slope		
significant main effects:		
ROI	F(3) = 3.23	p = 0.035
significant interactions:		
none		
not significant:		
condition	F(1) = 0.036	p = 0.85
ROI x condition	F(3) = 0.45	p = 0.72

fit line intercept		
significant main effects:		
condition	F(1) = 9.08	p = 0.012
significant interactions:		
none		
not significant:		
ROI	F(3) = 2.80	p = 0.055
ROI x condition	F(3) = 0.58	p = 0.63

Supplementary Table 3. Results of three-way repeated measures ANOVA with factors of ROI (IOG-/pFus-/mFus-/pSTS-faces), hemisphere (right/left), and condition (upright/inverted) on pRF estimates using a linear (non-compressive) pRF model (size = σ) in bilateral face-selective areas (N=12, related to **Supplementary Figure 7**).

center position Y estimate		
significant main effects:		
condition	$F(1,10) = 13.43$	$p = 0.0044$
significant interactions:		
none		
not significant:		
hem	$F(1,10) = 3.65x10^{-5}$	$p = 0.99$
ROI	$F(3,30) = 1.48$	$p = 0.24$
hem x ROI	$F(3,30) = 0.29$	$p = 0.83$
hem x condition	$F(1,10) = 4.27$	$p = 0.066$
ROI x condition	$F(3,30) = 1.49$	$p = 0.24$
hem x ROI x condition	$F(3,30) = 0.73$	$p = 0.54$

size estimate (σ)		
significant main effects:		
ROI	$F(3,30) = 8.01$	$p = 4.5x10^{-4}$
condition	$F(1,10) = 45.36$	$p = 5.1x10^{-5}$
significant interactions:		
none		
not significant:		
hem	$F(1,10) = 0.24$	$p = 0.64$
hem x ROI	$F(3,30) = 2.34$	$p = 0.094$
hem x condition	$F(1,10) = 0.0049$	$p = 0.95$
ROI x condition	$F(3,30) = 2.55$	$p = 0.074$
hem x ROI x condition	$F(3,30) = 0.11$	$p = 0.95$

center position X estimate		
significant main effects:		
hem	$F(1,10) = 442.09$	$p < 1.0x10^{-8}$
significant interactions:		
hem x ROI	$F(3,30) = 7.73$	$p = 5.7x10^{-4}$
not significant:		
ROI	$F(3,30) = 0.87$	$p = 0.47$
condition	$F(1,10) = 0.47$	$p = 0.51$
hem x condition	$F(1,10) = 0.049$	$p = 0.83$
ROI x condition	$F(3,30) = 0.23$	$p = 0.87$
hem x ROI x condition	$F(3,30) = 0.97$	$p = 0.42$

gain estimate		
significant main effects:		
ROI	$F(3,30) = 7.09$	$p = 9.7x10^{-4}$
condition	$F(1,10) = 67.12$	$p = 9.6x10^{-6}$
significant interactions:		
none		
not significant:		
hem	$F(1,10) = 1.26$	$p = 0.29$
hem x ROI	$F(3,30) = 0.47$	$p = 0.71$
hem x condition	$F(1,10) = 1.76$	$p = 0.21$
ROI x condition	$F(3,30) = 1.80$	$p = 0.17$
hem x ROI x condition	$F(3,30) = 0.087$	$p = 0.97$

model R^2		
significant main effects:		
ROI	F(3,30) = 20.61	p =1.9x10^{-7}
condition	F(1,10) = 70.48	p =7.7x10^{-6}
significant interactions:		
ROI x condition	F(3,30) = 5.74	p = 0.0032
not significant:		
hem	F(1,10) = 0.011	p = 0.92
hem x ROI	F(3,30) = 0.36	p = 0.78
hem x condition	F(1,10) = 2.13	p = 0.18
hem x ROI x condition	F(3,30) = 1.17	p = 0.34

Supplementary Table 4. Results of two-way repeated measures ANOVA on the individual participants' (N=12) coverage density metrics in bilateral face-selective areas (related to **Figure 5**).

coverage density FWHM area		
significant main effects:		
ROI	$F(3) = 9.74$	$p = 9.5\times10^{-5}$
condition	$F(1) = 8.41$	$p = 0.014$
significant interactions:		
none		
not significant:		
ROI x condition	$F(3) = 1.61$	$p = 0.21$

center of mass Y position		
significant main effects:		
ROI	$F(3) = 3.10$	$p = 0.040$
condition	$F(1) = 7.21$	$p = 0.021$
significant interactions:		
none		
not significant:		
ROI x condition	$F(3) = 0.39$	$p = 0.76$

center of mass X position		
significant main effects:		
none		
significant interactions:		
none		
not significant:		
ROI	$F(3) = 0.18$	$p = 0.91$
condition	$F(1) = 0.25$	$p = 0.63$
ROI x condition	$F(3) = 0.052$	$p = 0.98$

Supplementary References

1. Kay, K. N., Weiner, K. S. & Grill-Spector, K. Attention Reduces Spatial Uncertainty in Human Ventral Temporal Cortex. *Current Biology* **25**, 595–600 (2015).
2. Gomez, J., Natu, V., Jeska, B., Barnett, M. & Grill-Spector, K. Development differentially sculpts receptive fields across early and high-level human visual cortex. *Nat Comms* **9**, 788 (2018).
3. Finzi, D., Gomez, J., Nordt, M., Rezai, A. A., Poltoratski, S., & Grill-Spector, K. Differential spatial computations in ventral and lateral face-selective regions are scaffolded by structural connections. *Nat Comms* **12**, 2278 (2021).
