# Supplementary material for: Holistic face recognition is an emergent phenomenon of spatial processing in face-selective regions
Source: Nat Commun. 2021 Aug 6;12:4745. doi: 10.1038/s41467-021-24806-1 (PMC8346587; doi:10.1038/s41467-021-24806-1)
Supplement: Supplementary file 3 — Reporting Summary [file 41467_2021_24806_MOESM3_ESM.pdf]

nature research

Corresponding author(s): Sonia Poltoratski

Last updated by author(s): June 2, 2021

Reporting Summary

Nature Research wishes to improve the reproducibility of the work that we publish. This form provides structure for consistency and transparency in reporting. For further information on Nature Research policies, see our Editorial Policies and the Editorial Policy Checklist.

Statistics

For all statistical analyses, confirm that the following items are present in the figure legend, table legend, main text, or Methods section.

n/a	Confirmed	
☐	☒	The exact sample size (n) for each experimental group/condition, given as a discrete number and unit of measurement
☐	☒	A statement on whether measurements were taken from distinct samples or whether the same sample was measured repeatedly
☐	☒	The statistical test(s) used AND whether they are one- or two-sided<br>*Only common tests should be described solely by name; describe more complex techniques in the Methods section.*
☐	☒	A description of all covariates tested
☐	☒	A description of any assumptions or corrections, such as tests of normality and adjustment for multiple comparisons
☐	☒	A full description of the statistical parameters including central tendency (e.g. means) or other basic estimates (e.g. regression coefficient) AND variation (e.g. standard deviation) or associated estimates of uncertainty (e.g. confidence intervals)
☐	☒	For null hypothesis testing, the test statistic (e.g. F, t, r) with confidence intervals, effect sizes, degrees of freedom and P value noted<br>*Give P values as exact values whenever suitable.*
☒	☐	For Bayesian analysis, information on the choice of priors and Markov chain Monte Carlo settings
☒	☐	For hierarchical and complex designs, identification of the appropriate level for tests and full reporting of outcomes
☒	☐	Estimates of effect sizes (e.g. Cohen's d, Pearson's r), indicating how they were calculated

Our web collection on statistics for biologists contains articles on many of the points above.

Software and code

Policy information about availability of computer code

Data collection	Stimulus presentation and response collection was implemented using MATLAB (R2019a, https://www.mathworks.com/products/matlab.html) and Psychtoolbox (3, http://psychtoolbox.org).
Data analysis	fMRI data was preprocessed following a standard pipeline using FSL (5.0, https://fsl.fmrib.ox.ac.uk/fsl/fslwiki/Fslutils) and mrVista (https://github.com/vistalab) tools. We used the SPM (8; http://www.fil.ion.ucl.ac.uk/spm) difference-of-gammas hemodynamic response function (HRF) in estimating Beta values of response amplitude. Subsequent data analyses were carried out using custom Matlab scripts; code to reproduce all reported statistics and figures is publically available at github.com/VPNL/invPRF. Additionally, binary silhouette masks used for the pRF model fitting were created using Adobe Photoshop 2020.

For manuscripts utilizing custom algorithms or software that are central to the research but not yet described in published literature, software must be made available to editors and reviewers. We strongly encourage code deposition in a community repository (e.g. GitHub). See the Nature Research guidelines for submitting code & software for further information.

Data

Policy information about availability of data

All manuscripts must include a data availability statement. This statement should provide the following information, where applicable:

- Accession codes, unique identifiers, or web links for publicly available datasets
- A list of figures that have associated raw data
- A description of any restrictions on data availability

Data from the neuroimaging study,ehavioral eye tracking study, and simulations is publicly available at github.com/VPNL/invPRF. Source data are provided with this paper: data for every figure and statistical result in the manuscript are released as Matlab .mat files, which can be read as HDF5 files in Python. A README.md file describes the organization of the data in the repository. Raw neuroimaging data is available under restricted access for the data privacy of our participants; access

can be obtained by request of the authors.

Field-specific reporting

Please select the one below that is the best fit for your research. If you are not sure, read the appropriate sections before making your selection.

☒ Life sciences ☐ Behavioural & social sciences ☐ Ecological, evolutionary & environmental sciences

For a reference copy of the document with all sections, see nature.com/documents/nr-reporting-summary-flat.pdf

Life sciences study design

All studies must disclose on these points even when the disclosure is negative.

Sample size	Population receptive fields (pRFs) are modeled within each subject and voxel independently. The sample size (number of pariticipants) was determined from previous studies employing similar pRFs methodologies in the visual system which use data from a range of 3-20 participants (Dumoulin & Wandell 2008; Kay, Weiner, & Grill-Spector 2015; Le et al., 2017; Witthoft et al., 2016; Klein, Harvey, & Dumoulin, 2014; Poltoratski & Tong, 2020). Here we collected data from 13 subjects. One subject was excluded due to excessive motion (see below) and we report data from 12 subjects. The number of voxels per participant per area is specific to the size of their independently-defined cortical visual areas, determined empirically from independent scans.
Data exclusions	One subject's data was excluded from the fMRI experiment due to excessive motion during scanning (>4mm). Three subjects' data were excluded from the behavioral eye tracking study due to failure to fixate (>25% of all trials). Both criteria were established in advance.
Replication	We fit an independent pRF model to the data of each voxel of several visual areas in each individual. This process was repeated independently in 12 participants. Additionally, all data were fit using two independent pRF models (CSS, or compressive spatial summation, and linear). Results replicate across pRF models and are consistent across individuals.
Randomization	All conditions were tested within-participants, so random allocation into groups was not necessary.
Blinding	Group allocation was not performed; thus, blinding was not necessary.

Reporting for specific materials, systems and methods

We require information from authors about some types of materials, experimental systems and methods used in many studies. Here, indicate whether each material, system or method listed is relevant to your study. If you are not sure if a list item applies to your research, read the appropriate section before selecting a response.

Materials & experimental systems

n/a	Involved in the study	
☒	☐	Antibodies
☒	☐	Eukaryotic cell lines
☒	☐	Palaeontology and archaeology
☒	☐	Animals and other organisms
☐	☒	Human research participants
☒	☐	Clinical data
☒	☐	Dual use research of concern

Methods

n/a	Involved in the study	
☒	☐	ChIP-seq
☒	☐	Flow cytometry
☐	☒	MRI-based neuroimaging

Human research participants

Policy information about studies involving human research participants

Population characteristics	Thirteen participants (6 women) ages 20-31 participated in the experiment. Nine participants identified as white/Caucasian, 1 as Asian, 1 as Black, and 2 as mixed-race (Hispanic/white and Asian/white).
Recruitment	Participants were recruited from the Stanford University community and participated in one experimental scanning session, one retinotopic/localizer scanning session, and one behavioral eye-tracking session. We do not expect biases in participant recruitment to meaningfully impact these results. Several participants were fMRI researchers within the Stanford Psychology Department, which may have increased the data quality (insofar as it is dependent on participant motion and alertness) relative to a random population sample. Only author SP was aware of the specific hypotheses of the study while participating. We confirmed via additional analysis that the results are unchanged via exclusion or replacement of her data.
Ethics oversight	The Stanford University Institutional Review Board approved all aspects of this study.

Note that full information on the approval of the study protocol must also be provided in the manuscript.

Magnetic resonance imaging

Experimental design

Design type	Event-related (randomized 4s trials) pRF mapping task.
Design specifications	The main experiment consisted of 8-10 runs of pRF mapping and lasted approximately 90 minutes. Each pRF mapping run lasted 282 seconds, and presented face images at 25 randomized spatial positions to span the visual field over time. Faces were presented in 4s trials of faces (50 trials, 25 positions x upright/inverted) and blank periods (10 trials), which were randomly ordered for each run and each participant. Each run began and ended with an additional 16s blank period.
Behavioral performance measures	Participants performed a challenging RSVP letter detection task at the central fixation point throughout each mapping run. Performance was monitored via recorded keypresses, and fixation was monitored via eyetracking in the scanner.

Acquisition

Imaging type(s)	functional MRI
Field strength	3T
Sequence & imaging parameters	Functional T2* weighted pRF mapping runs were collected using a 32-channel headcoil, single-shot EPI, with a voxel resolution of 2.4mm isotropic and a TR of 2s (FOV = 192, TE = 30ms, flip angle = 77deg).
Area of acquisition	Twenty-eight slices were prescribed parallel to the parieto-occipital sulcus to cover each participant's occipital and ventral temporal lobes.

Diffusion MRI ☐ Used ☒ Not used

Preprocessing

Preprocessing software	Anatomical MRI was segmented using FreeSurfer v5.3c; Manual corrections of the FreeSurfer segmentation were done in ITKGray.<br>Functional MRI was motion corrected, aligned to each subject's anatomical MRI, high pass filtered to remove drift, and slice-time corrected. No spatial smoothing was applied.
Normalization	No normalization was applied; all data were analyzed in the native brain space of each participant
Normalization template	The data were not normalized.
Noise and artifact removal	Between- and within-scan motion correction was applied, as well as high-pass filtering to remove fMRI drift.
Volume censoring	No volume censoring was done.

Statistical modeling & inference

Model type and settings	Timecourse data for each voxel was fit with a general linear model (GLM) to separate responses in the two stimulus conditions. Subsequently, the compressive spatial summation CSS pRF model (Kay 2013; 2015) was fit independently in each condition (upright/inverted) in each voxel.<br>To evaluate the strength of effects across subjects, we used repeated-measures ANOVAs over subject-wise means of estimated model parameters. Additional quantification of model parameter estimates (size x eccentricity) was done via bootstrapped linear regression fitting.
Effect(s) tested	Initial evaluation of model estimates were done with a 3-way repeated measures ANOVA including factors of hemisphere, region of interest (ROI), and experimental condition. As there were no significant effects of hemisphere on any parameter estimate, subsequent analyses combined data across hemisphere in all ROIs.

Specify type of analysis: ☐ Whole brain ☒ ROI-based ☐ Both

Anatomical location(s)	Functional regions of interest (ROIs) in the face network and early visual cortex were defined from independent fMRI data in the native brain space of each subject. Face-selective ROIs were defined from a localizer scan (as in Stigliani et al, 2015); we label ROIs by selectivity and anatomical location. Retinotopic ROIs from a traveling wave experiment using bars filled with brightly-colored cartoon images (as in Finzi et al, 2020).
Statistic type for inference (See Eklund et al. 2016)	Voxel-wise; Functional ROIs were defined based on localizer scan with a face selectivity exceeding a t ≥ 2.3 threshold. Within these ROIs, we report pRF model parameters for each voxel in which the pRF model explained at least 20% of their variance during the face pRF mapping experiment in each of the upright and inverted mapping conditions.
Correction	None

Models & analysis

n/a	Involved in the study
☒	☐ Functional and/or effective connectivity
☒	☐ Graph analysis
☐	☒ Multivariate modeling or predictive analysis

Multivariate modeling and predictive analysis	The parametric CSS pRF model was fit to voxelwise pRF parameters to response amplitudes (GLM beta estimates) to face-stimuli (upright/inverted) in each of 25 locations in the visual field. This model estimates for each voxel, the parameters of the pRF: gain, X, Y, sigma, n. Model fitting was performed using nonlinear optimization (MATLAB Optimization Toolbox) via the Levenberg–Marquardt algorithm. Model fits were evaluated based on the coefficient of determination (R^2).
